# Association between climate indicators and hay fever incidence in children and adolescents in Freiburg, Germany

**DOI:** 10.3389/fpubh.2025.1587767

**Published:** 2025-06-04

**Authors:** Trang Dao-Siebel, Jakob Holstiege, Kathrin Graw, Christoph Müller, Andreas Matzarakis, Roxana Halbleib, Evelyn Lamy

**Affiliations:** ^1^Faculty of Economics and Behavioral Sciences, University of Freiburg, Freiburg, Germany; ^2^Research of Environment-Related Mechanisms of Action on Health, Medical Faculty, University of Augsburg, Augsburg, Germany; ^3^Central Research Institute of Ambulatory Health Care in Germany, Berlin, Germany; ^4^German Meteorological Service, Research Centre Human Biometeorology, Freiburg, Germany; ^5^Center for Pediatrics and Adolescent Medicine, Faculty of Medicine, University of Freiburg, Freiburg, Germany; ^6^Environmental Meteorology, Department of Earth and Environmental Sciences, Faculty of Environment and Natural Resources, University of Freiburg, Freiburg, Germany; ^7^Department of Epidemiology and Healthcare Atlas, Democritus University of Thrace, Komotini, Greece

**Keywords:** allergic rhinitis, children and adolescents, climate change, generalized additive models, hay fever, incidence

## Abstract

**Background:**

Allergic conditions including hay fever are a sentinel measure of environmental impact on human health in early life. In this study we investigated the association between climate indicators and allergic rhinitis (hay fever) incidence in children and adolescents in Freiburg im Breisgau (Germany), as a representative study site for an urban German environment.

**Methods:**

Data on climate indicators and hay fever incidence in children and adolescents in the period 2013 to 2021 were implemented within the free software environment for statistical computing R using generalized additive Gamma family models.

**Results:**

Our results from all “seasonal”, “non-seasonal”, and “single-factor” models could not support the associations between the hay fever incidence and the precipitation as well as the concentrations of PM_10_, NO_2_, and O_3_ in Freiburg. However, they indicated statistically significant associations with temperature, and wind speed at the 5% level. The hay fever incidence was highest, as the temperature was between 4–6°C, and 10–17°C, and the wind speed was between 2.0 and 2.1m/s.

**Conclusions:**

This knowledge could be of relevance for the choice of patient treatment procedure in Freiburg, as the symptoms of a cold or flu can easily be mistaken for an allergy, especially in the cold season.

## 1 Introduction

Climate change is one of the most difficult and complex challenges facing the world, causing numerous environmental changes such as rising temperatures, changes in precipitation, more intense and frequent extreme events such as heat waves, droughts, storms (1), and worsening air quality (2). All these climate change indicators have increasingly negative impacts on humans and natural systems (3, 4).

Allergic conditions are a sentinel measure of environmental impact on human health in early life. In Germany, approximately 34% of the population is affected by at least one allergic disease in their lives, and half of the population has shown allergic sensibilization (5). Respiratory allergies are among the most common respiratory diseases and represent a major contributor to both direct and indirect healthcare costs. Allergic rhinitis or hay fever is the predominant prevalent type of respiratory allergy and known for affecting the quality of life. Studies disclosed that in Germany 15% of adults (6), and 9.9% of children and adolescents (7, 8) suffered from hay fever. Consequently, treatment costs for only pollen allergy sufferers amount to around 240 million Euros on average every year (9).

There is a notable amount of literature on the association between hay fever and climate indicators, but the outcomes are often mixed and sometimes contradictory. Regarding temperature and extreme events, several studies demonstrated, that both, heat and cold, can significantly increase the risk of hay fever (10–15). In contrast, Hsieh et al. reported a negative relationship between temperature and rhinitis cases (16), while Wang et al. found a positive association (17). Interestingly, until now, very few studies report on the effect of wind speed and precipitation on hay fever. The existing studies on this topic have produced conflicting results, for instance Wang et al. found that wind speed is negatively associated with hay fever (17), whereas Lou et al. found a positive association (18). Similarly, the relationship between precipitation and allergic rhinitis has also yielded conflicting findings. A study by Wang et al. reported a positive association between precipitation and hay fever (17), whereas a study by Peternel et al. found that the more it rains the less allergen exposure occurs (19). Therefore, a negative association between precipitation and hay fever incidence could be expected.

Additionally, an increasing concentration of ground-level ozone (O_3_) has also been considered as a climate change indicator. O_3_ is a respiratory irritant and can worsen hay fever symptoms as demonstrated in some studies (20–24). However, also no significant association was found between the allergic rhinitis and O_3_ (25–27). The formation (and degradation) of O_3_ correlates well with nitrogen oxide (NO_2_) levels (28, 29). Hence, it is important to pay attention to the relationship between NO_2_ and hay fever, as well. Higher NO_2_ concentration may be associated with lower pollen quantity due to the negative effects of NO_2_ on either the development of plants or on their blossoming and pollen production (30–32). *In vitro*, NO_2_ can cause a general drop in the germination and allergenic protein content of exposed pollen grains (33, 34). Thus, a negative relationship between pollen season and NO_2_ could be assumed. While Burte et al. and Gehring et al. could not find evidence of association between NO_2_ and rhinitis (35, 36), others reported that the exposure to NO_2_ negatively impacted the respiratory system and exacerbated the severity of the allergic airway inflammation (37, 38). Similar to the gaseous air pollutants, contrasting results about association between hay fever and PM_10_ levels were also found. While Kang et al. indicated that no significant correlations were observed between changes in the PM_10_ concentration and allergic symptom scores (39), Pénard-Morand et al. and Puklová et al. found that PM_10_ positively associated with both lifetime and current allergic rhinitis (40, 41).

All these contradictory results call for further research to better understand these relationships and develop effective prevention and intervention strategies, especially in the context of climate change. Furthermore, the effects of climate change indicators are multifaceted, with each geographic region having its own characteristics. Specifically, we clarified that children with hay fever have an elevated risk of developing asthma (42), which has been associated with long-term reductions in adult lung function (43). Furthermore, children are potentially more susceptible to environmental allergens and pollutants due to their higher oxygen consumption per unit of body weight and the immaturity of their respiratory and immune systems (44). Additionally, as children spend more time in outdoor environments, their exposure to outdoor environmental factors is likely to be greater (44).

Against this background, the present study investigated the association between different climate change indicators and hay fever in children and adolescents in the middle European city Freiburg im Breisgau (Germany) using quarterly incidence data from 2013 to 2021 in the age group 0–17 years. To the best of our knowledge, there are no comparable studies for Germany covering the last decade.

## 2 Materials and methods

### 2.1 Study area

**Figure 1:**
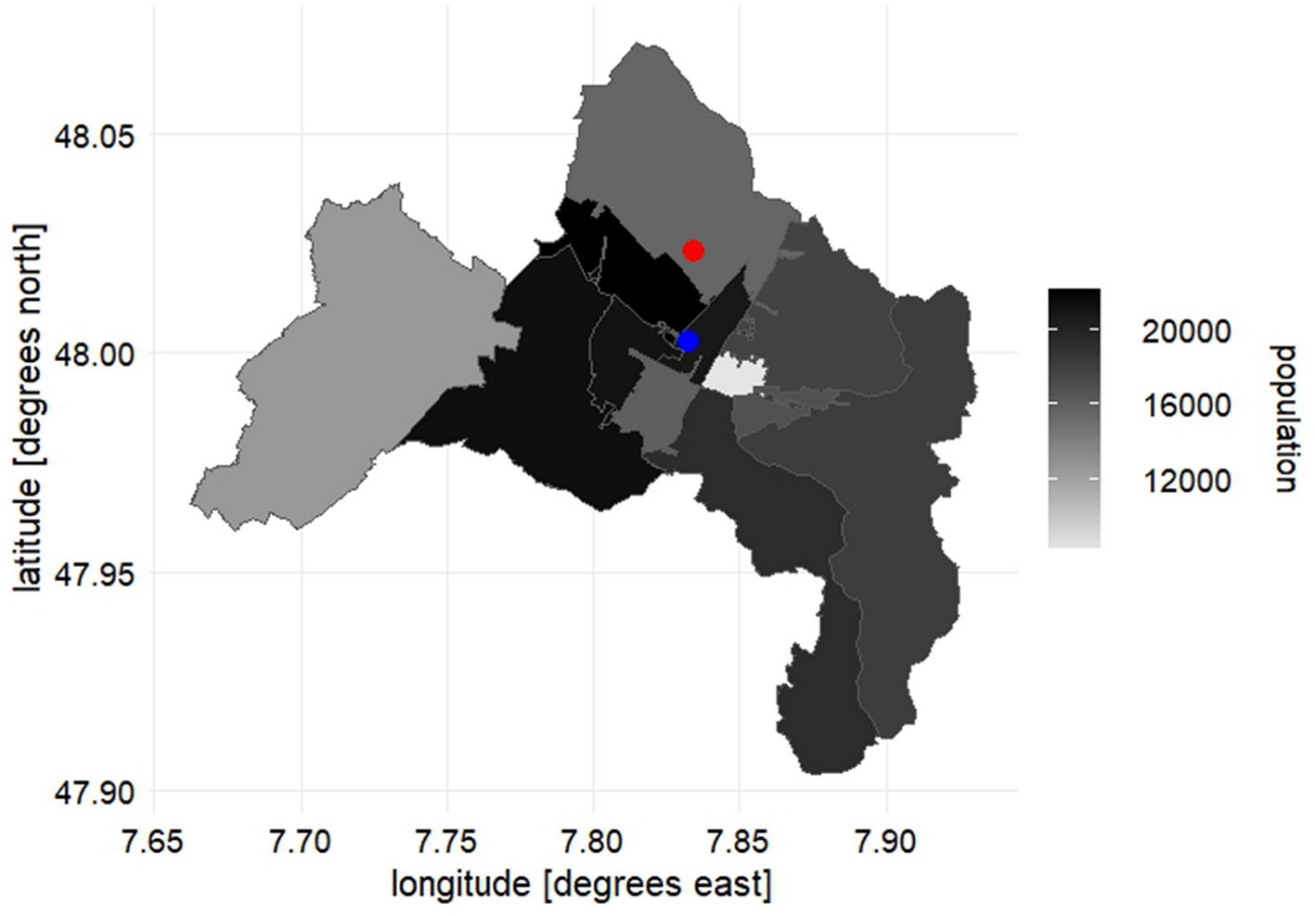
An overview map of Freiburg im Breisgau. The population decreases gradually across regions from black to gray. The red and blue points are the observation stations of the meteorological parameters and the air pollutants, respectively.

Freiburg im Breisgau (Germany) was chosen as a representative study site for an urban setting, because of its unique characteristics. The city has attracted global attention for its sustainable urban development, successes in green, low-carbon economy, mobility, energy, and land use planning (45). Freiburg is located on the western edge of the Black Forest in the Upper Rhine Valley in southwestern Germany, and it is well known as one of the warmest areas in the country. It has a size of 15,307 ha (46), a population of 234,442 people (47), and a high population density of 1,511/km^2^. An overview map of the study area can be seen from Figure 1 (for detailed material and methods consult the Supplementary material S1).

### 2.2 Data of climate indicators

For this study, several climate indicators, such as daily mean temperature at 2 m above ground level (°C), daily total precipitation (mm), and daily mean wind speed (m/s) of station no. 1443 Freiburg, sampled from 2007 to 2021, have been provided by the German Meteorological Service (Deutscher Wetterdienst DWD, 2022). Hourly data for ozone O_3_ (μg/m^3^) and nitrogen dioxide NO_2_ (μg/m^3^) from 2007 to 2021, as well as particulate matter of aerodynamic diameter less than 10 μm PM_10_ (μg/m^3^) from 2013 to 2021 for the Freiburg DEBW084 station, provided by Federal Environment Agency, have also been selected. These stations are typical for urban residential areas away from the major roads (Figure 1). For the studied period, the measurement of particulate matter of aerodynamic diameter <2.5 μm PM_2.5_ in the study area could not be fully accounted for, as the station measuring PM_2.5_ was not established until 01.01.2019.

### 2.3 Respiratory allergy data

Quarterly incidence of hay fever per 1,000 children and adolescents at risk aged 0–17 years in Freiburg, from 2013 until 2021, were provided by the Central Research Institute of Ambulatory Health Care (Germany). Calculations were done using outpatient claims data of the German Statutory Health Insurance according to § 295 of the German Code of Social Law (Fünftes Sozialgesetzbuch, SGB V). The methodology applied to estimate hay fever incidence (HFI) based on insurance claims has been described elsewhere (7). In brief, the occurrence of newly diagnosed hay fever was assessed in annual cohorts of patients in the age group 0–17 years, who were observable in the year of reporting, and in the three previous years, or who were born in this four-year period. The children and adolescents, who did not receive a diagnosis of hay fever during the pre-observation period were eligible for inclusion into the population at risk. Incident cases were identified using diagnoses coded according to the International Statistical Classification of Diseases and Related Health Problems, 10th revision, German modification (ICD-10-GM-codes: 30.1 for hay fever due to pollen, J30.2 for other seasonal hay fever). New hay fever cases were defined as the first occurrence of disease-specific diagnostic codes in the respective populations at risk, together with the disease modifier “assured” after a diagnosis-free period of 3 years and repeated coding at least once in the following four quarters after the index quarter. Quarterly cumulative incidence was calculated per 1,000 children and adolescents under risk. Demographic details for the population at risk of hay fever are provided in the Supplementary material S2.

Quarter one (Q1) refers to the months of January, February, and March; quarter two (Q2) to the months of April, May and June. Quarter three (Q3) refers to the months of July, August and September, and quarter four (Q4) to the months of October, November and December.

### 2.4 Data analysis and method description

First, we analyzed the time evolution of the variables. Since the variables had been collected at different frequencies, we had to work with the lowest frequency available, i.e., the quarterly data. For this purpose, we aggregated the data at the higher frequency to the quarterly level by averaging or summing up by using functions in Microsoft Excel © Version 2301. In the next steps, methods and packages within the free software environment for statistical computing R (Version 4.2.2, Germany) were used. We computed correlations among the climate indicators in order to understand their dependencies and potentially multicollinearity that may lead to confounding effects and, thus, poor estimates of their association with the HFI. We presented in this paper results on the Spearman rank correlations, as they are less sensitive to outliers and the normality assumption. However, for robustness check, we also implemented the classical Pearson correlation. The results were consistent with those obtained from the Spearman correlation analysis, both in terms of direction and magnitude. Therefore, we did not include them in the manuscript; however, they are available from the authors upon request. Next, we implemented generalized additive models (GAMs) with the Gamma distribution family [the function “gam()” from the package mgcv] to analyse the associations between the climate indicators and the hay fever. This is a very general methodological framework that can capture linear, monotonic or more complex nonlinear relationships, depending on the way each variable responds to changes in the dependent variables (48, 49).

Here we first computed models named “seasonal”, which focused on studying the multivariables' effects on the HFI with time seasonality controlled for. As described below, we work with semi-annual seasonality. Here, the seasonality was added in the model as a categorical variable including two categories Q1Q4 and Q2Q3, since Q1 and Q4 shared closely similarities, as are the other two quarters. We applied cubic regression splines smooth function for the temperature, NO_2_ and O_3_, since they are useful for fitting models with seasonal patterns (50, 51). For the other variables, we kept the thin plate regression splines, which are the default smooth (52). Based on environmental theory and previous studies, there may be interactions between PM_10_, NO_2_, O_3_ and precipitation (53–56), as well as wind speed (57, 58), between NO_2_ and O_3_ (59), and between temperature and wind speed (60). Therefore, we also considered these interactions in our models by using tensor product smooths function (50, 52). Since categorical data, here for the seasonality, in GAMs are treated as a linear term without smoothing (48), we applied the seasonality in linear manner.

For the robustness check, we assessed additional models assigned here as “single-factor” and “non-seasonal”. Due to the limited data, we needed to balance the effects of adding further variables in the regression: more relevant variables added, less bias in the estimates, but lower standard errors; less variables added, more bias, but higher standard error. Therefore, for the “single-factor” models we retained the seasonality and one climate indicator as the only two predictors at a time. For the non-seasonal models, we fitted the joint effect of the predictors, and excluded the seasonality, which allow us to examine contribution of seasonality by comparing the results.

In all models, we computed functions “gam.check()”, and “AIC()” to achieve optional fitness. The statistically significant level has been chosen at upmost 5%. We estimated the quarterly HFI using the “predict” function for all chosen predictors, with setting the other predictors to their medians (61). Finally, we applied the package “ggplot2” to visualize the significant associations.

## 3 Results

### 3.1 Variation in hay fever incidence

**Figure 2:**
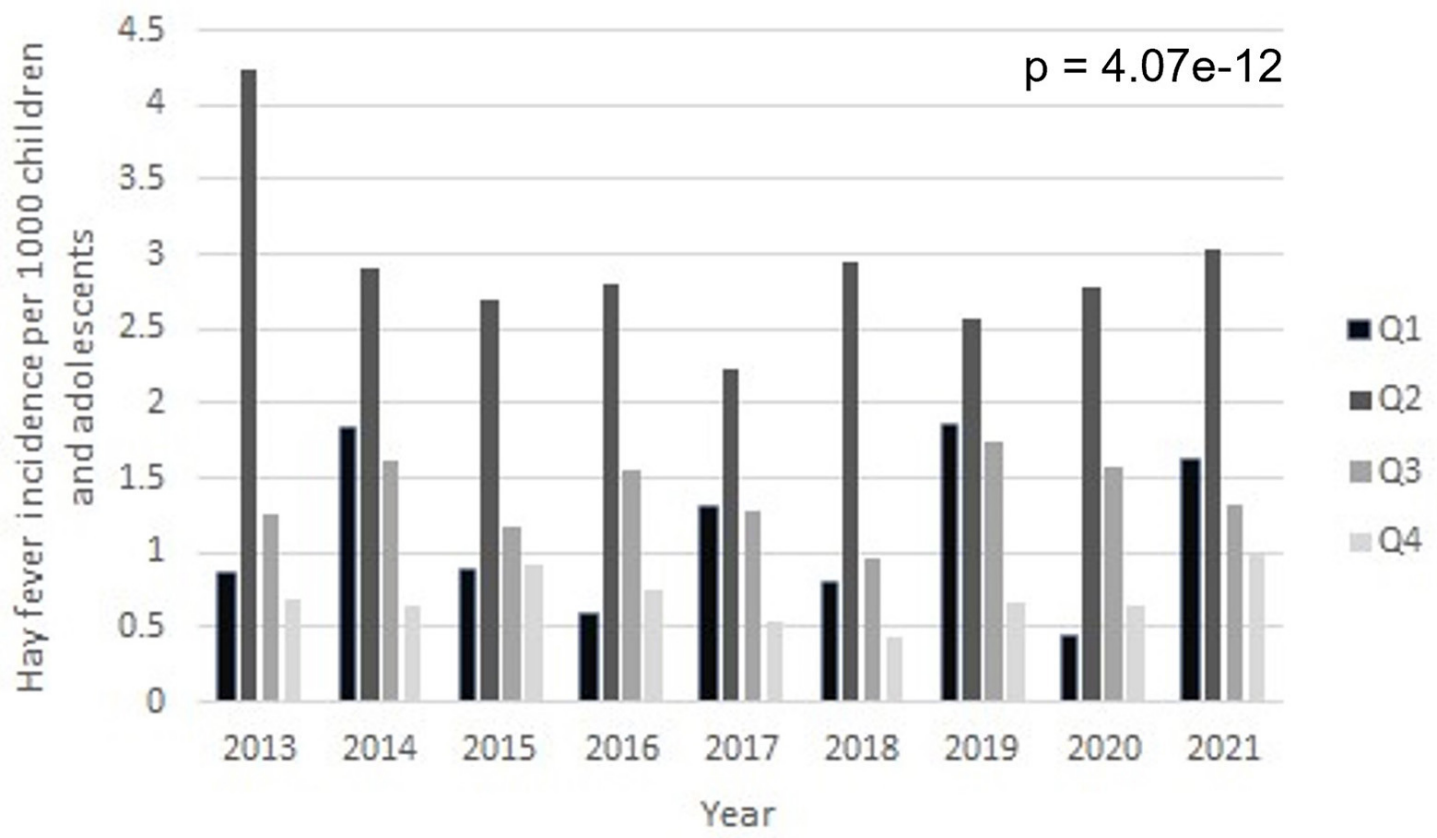
Quarterly HFIs for Freiburg in the period between 2013 and 2021. Bars are HFI data corresponding to Q1-4.

Figure 2 displays the HFI's levels across years and quarters. No significant changes in the quarterly and yearly HFI between 2013 and 2021 were observed, which might be due to the relatively short window. HFI was significantly higher in Q2 than in the other three quarters over all 9 years investigated (*p* = 4.07e-12).

### 3.2 Variation in climate indicators

**Figure 3:**
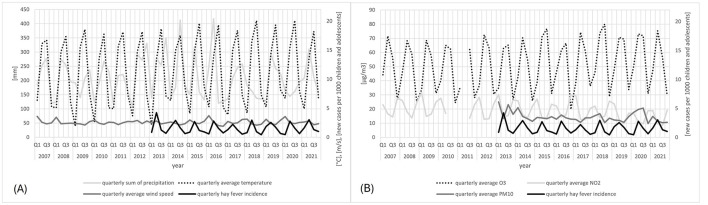
Time series of the quarterly HFI from 2013 to 2021 and climate indicators **(A)** meteorological parameters, i.e. temperature, precipitation, and wind speed; **(B)** PM_10_ from 2013 to 2021 and gaseous air pollutants (O_3_, NO_2_) from 2007 to 2021. The gaps in the graphs are due to missing data.

Figures 3A, B provide the time series of quarterly observations of the climate indicators as explanatory variables and the HFI as the dependent variable. Quarterly variations in hay fever frequency corresponded to changes in the quarterly temperature (Figure 3A), the quarterly wind speed (Figure 3A), and the quarterly O_3_ (Figure 3B), whereas they varied inversely with the quarterly NO_2_ (Figure 3B). No correspondences between other indicators were observed.

**Figure 4:**
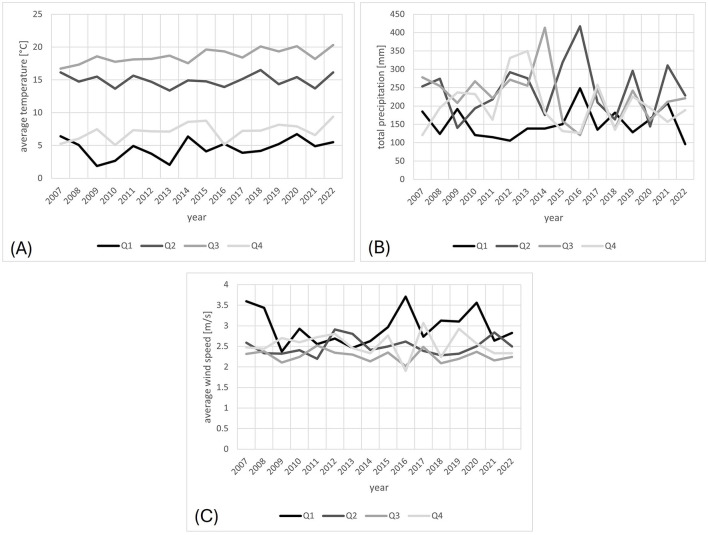
**(A)** Average temperature, **(B)** total precipitation, and **(C)** average wind speed based on the data in Freiburg from 2007 and 2021, given per quarter. The lines are quarterly average temperature, total precipitation, and wind speed in **(A–C)**. The color intensity fades gradually in the order of the quarters, from 1 to 4.

To discover possible changes in climate indicators in the individual quarters of the study period associated with hay fever, we first visualized the meteorological parameters in Figure 4. In contrast to a stable average temperature throughout the years in Freiburg, a significant increase in temperature could be detected for Q3 and Q4, during the period 2007–2021 (*p* = 0.001 and 0.039, respectively). No significant changes in wind speed and precipitation were found.

**Figure 5:**
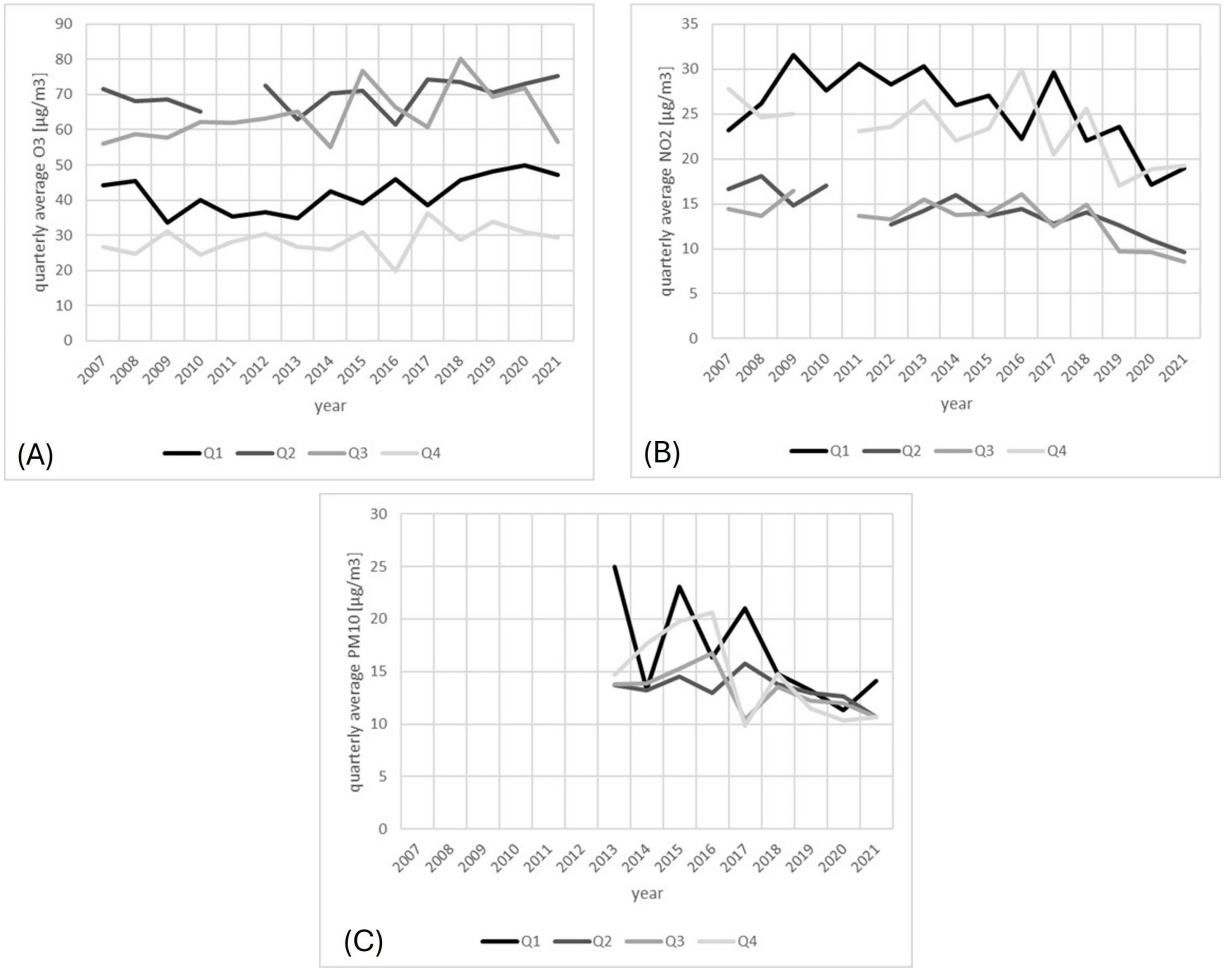
Average concentration of **(A)** O_3_, **(B)** NO_2_, and **(C)** PM_10_, based on quarterly data for Freiburg from 2007 and 2021. The gaps in the graphs are due to missing data, which however does not cover the period considered in our analysis. The color intensity fades gradually in the order of the quarters, from 1 to 4.

Next, we considered the temporal course of the most abundant air pollutants NO_2_, ground-level O_3_, and PM_10_. The NO_2_ and the PM_10_ showed significantly decreases over the observed years (*p* = 0.021 and 0.001, respectively), whereas the O_3_ concentration remained unchanged (Figure 5).

As one may observe from Figures 2–5, the variables at hand display quarterly seasonality effects. However, given that we have only a few numbers of observations per series (36), we needed to balance the effects of adding further variables in the regression, i.e. balance between reducing the bias in the estimates and increasing their standard errors. This trade-off is particularly important when dealing with such a small sample size. Therefore, we worked here with semi-annual seasonal variables, instead of quarterly.

### 3.3 Correlations between climate indicators

**Figure 6:**
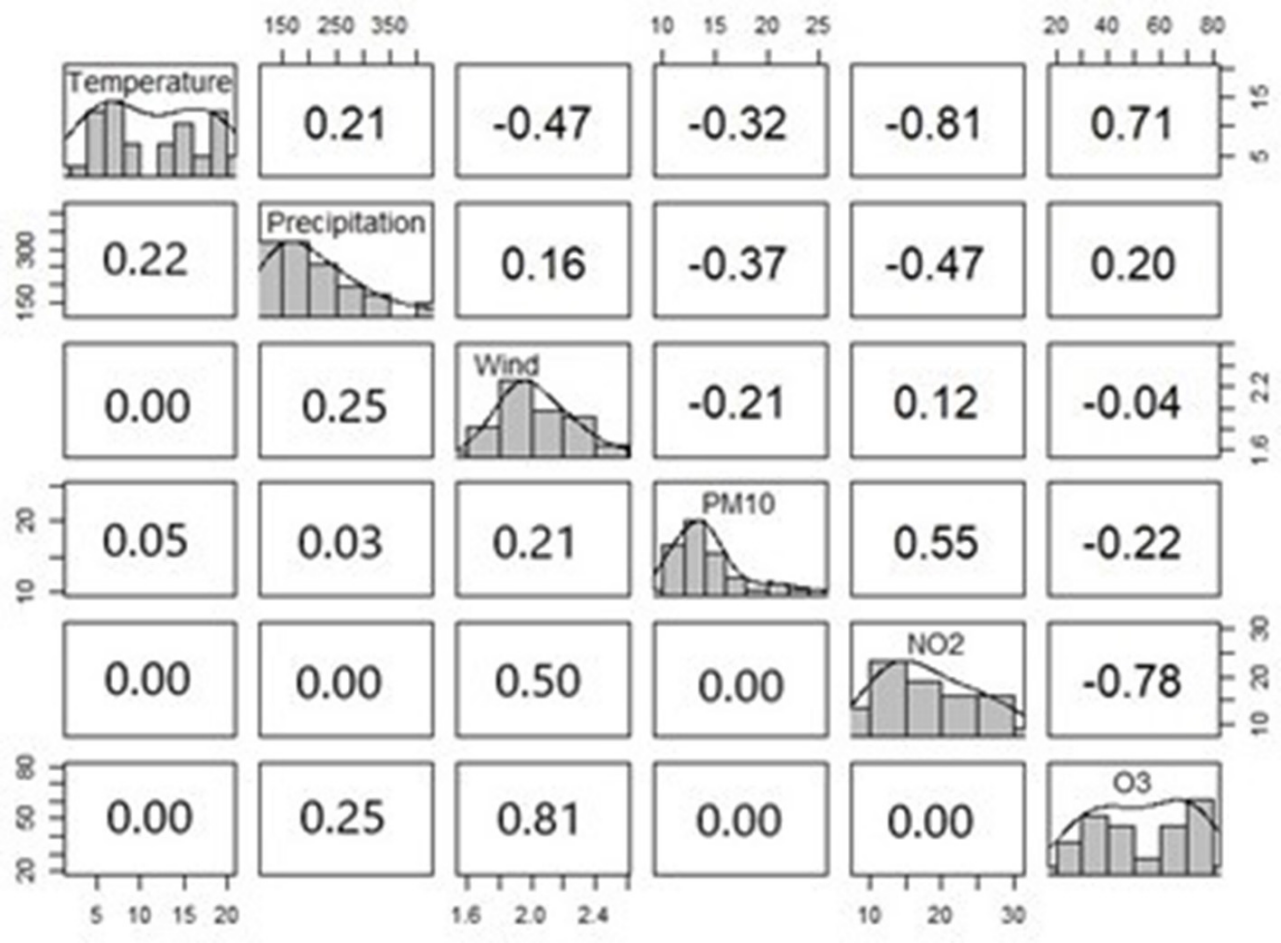
Spearman rank correlation among climate indicators based on quarterly data for Freiburg from 2013 and 2021. On the diagonal we provide, histograms and probability density shapes of the variables at hand. The upper triangular provides the correlations. The lower triangular provides the respective *p*-values of the correlations from the upper triangular.

In Figure 6, we present results from computing the Spearman rank correlation among the climate indicators.

The results given in Figure 6 align with the basic environmental principles: temperature clustered around two means due to seasoning effects, precipitation, PM_10_ and NO_2_ were right skewed, indicating a higher probability mass to lower values, while the O_3_ level seemed to be rather uniformly distributed, wind speed distributed normally. We also observed high correlations between the temperature and the O_3_ (positive 71%), as well as the NO_2_ (negative 81%). Correlations between the NO_2_ and the PM_10_ (positive 55%), as well as the O_3_ (negative 78%) have been also detected. The correlations among the other factors were smaller, and sometimes not statistically significant at 5% level. Accordingly, for our regression analysis, we considered temperature, precipitation, wind speed, and PM_10_ as predictors in a first step. Then, the effects of O_3_, and NO_2_ were considered, by successively replacing the temperature and PM_10_. Due to the limited data, we individually applied the interactions between each two indicators, which have been mentioned at the method description.

### 3.4 Outcome model

**Figure 7:**
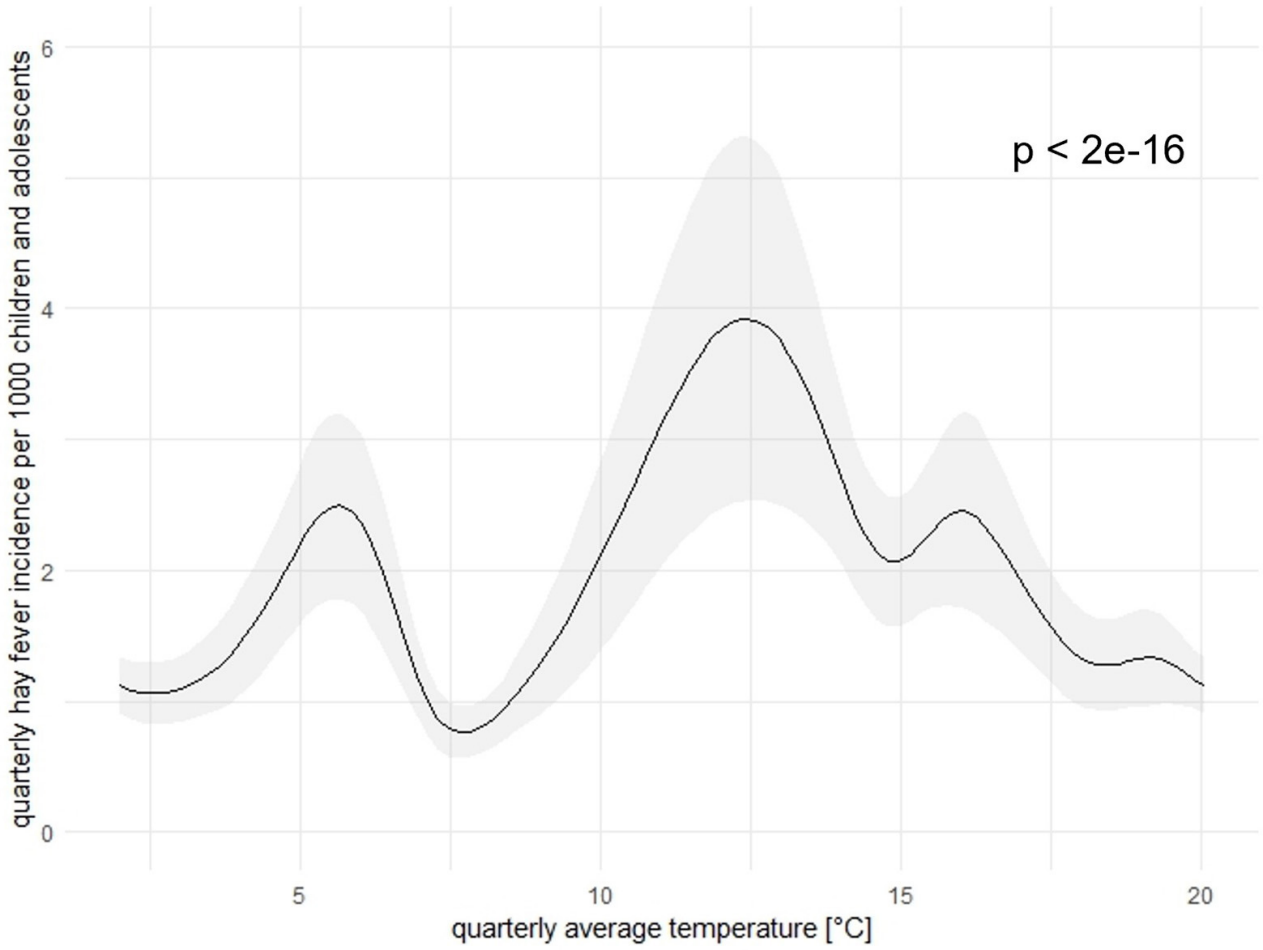
Association between the quarterly HFI and the quarterly average temperature [°C] in Freiburg in calendar quarter from 2013 to 2021. The black line represents the association, the gray shaded area represents 95%CI. To note, here we revealed the curve in the “seasonal” model, curves in the “single-factor” and the “non-seasonal” models are similar.

**Figure 8:**
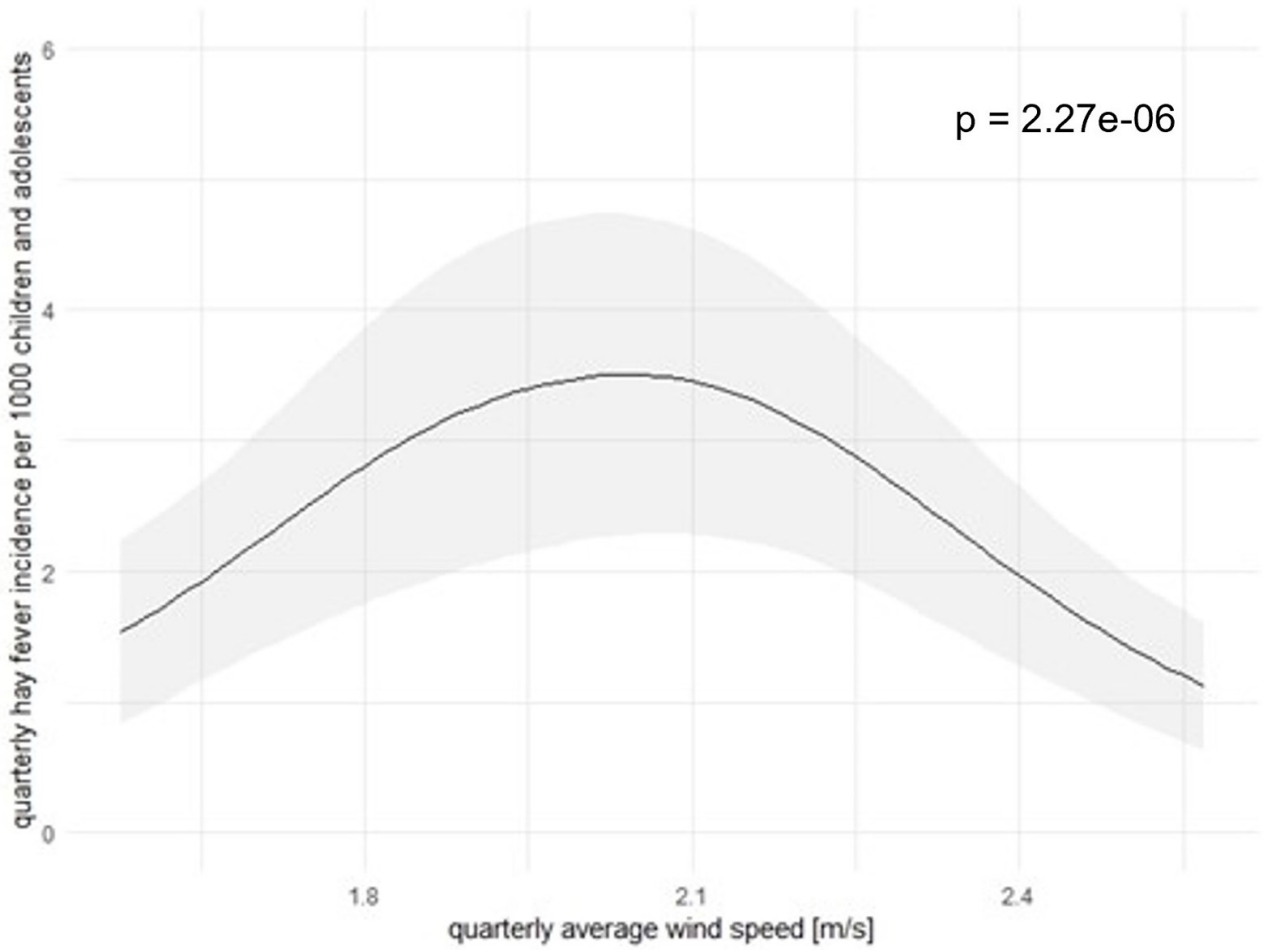
Association between HFI and average wind speed [m/s] in Freiburg in calendar quarter from 2023 to 2021. The black lines represent the associations between HFI and the average wind speed, the gray shaded area represents 95%CI. Here we also revealed the curve in the “seasonal” model, curves in the “single-factor” and the “non-seasonal” models are similar.

Results from our “seasonal”, “single factor”, and “non-seasonal” models provide the estimated associations between the HFI and the temperature (Figure 7), as well as the wind speed (Figure 8). The two associations were statistically significant at 5% significance level. The association between the temperature and the HFI varied in a larger scale than the one of the wind speed. In our “seasonal” and “non-seasonal” models, we also identified a significant relationship between the HFI and the O_3_. This may be caused by a confounding variable, i.e. the wind speed. When we excluded this variable in the models, O_3_ was no longer significant. Hence, we did not visualize this association here. For precipitation, PM_10_, NO_2_ as well as the interactions, we could not find any statistically significant association with the HFI at the 5% level. However, it is important to emphasize that the associations between the HFI and the interactions among temperature and wind speed, as well as the one among wind speed and O_3_ were significant at 9% and 6% level, respectively. These associations may be significant at 5% level in a larger dataset. Models' summaries can be found in Table 1.

**Table 1:** GAMs summaries for hay fever incidence.

**Models**	**Approximate significance of smooth terms**	**edf**	**Ref. df**	**F**	***p*-value**
Seasonal	s (Wind)	3.653	4.289	15.88	**2.27e-06**
	s (Temperature)	8.866	10.000	12.89	**<2e-16**
	s (Precipitation)	1.000	1.000	2.596	0.123
	s (PM_10_)	1.000	1.000	0.016	0.902
	s (NO_2_)	0.00019	8.000	0.000	0.401
	s (O_3_)	2.375	8.000	0.808	**0.0435**
	ti (Wind, PM_10_)	1.554	1.877	0.543	0.496
	ti (Wind, O_3_)	5.985	7.426	2.316	0.052
	ti (Wind, NO_2_)	1.000	1.000	1.376	0.250
	ti (Precipitation, PM_10_)	2.360	3.288	0.633	0.594
	ti (Precipitation, O_3_)	2.138	2.570	2.824	0.100
	ti (Precipitation, NO_2_)	1.000	1.000	3.106	0.092
	ti (NO_2_, O_3_)	2.059	2.418	1.172	0.254
	ti (Temperature, Wind)	2.900	3.514	2.253	0.086
	Seasonality	Different between the models			
Non seasonal	s (Wind)	3.771	4.62	11.564	**2.03e-05**
	s (Temperature)	8.437	10.00	27.078	**<2e-16**
	s (Precipitation)	1.8569823	2.266	0.861	0.553
	s (PM_10_)	1.959	2.388	1.489	0.231
	s (NO_2_)	0.0001793	8.000	0.000	0.382
	s (O_3_)	3.4584655	8.000	7.698	**1.07e-06**
Singel factor	s (Wind)	3.362	4.165	4.291	**0.006**
	s (Precipitation)	1.000	1.000	0.001	0.971
	s (PM_10_)	3.455	4.268	1.249	0.292
	s (Temperature)	4.135	8.000	3.199	**0.0004**
	s (NO_2_)	2.638e-05	8.000	0.000	0.829
	s (O_3_)	1.403	8.000	0.33	0.141
	Seasonality	Different between the models			

Statistically significant p-values are shown in bold.

The smoothed curve in Figure 7 indicated that the direction of the relationship between the HFI and the temperature changed midway through the range of the temperature. Overall, the curve shows optimal ranges of the temperature were between 4–6°C and 10–17°C. The curve started high, then increased rapidly to 2.5 new cases as the temperature is at 6°C. After that, the curve sloped downward, indicating a negative relationship. In other words, the more the temperature rose (up to 8°C), the more the HFI reduced. As the temperature rose from 8°C to 13°C, the curve was upward-sloping, showing a positive relationship. Here, the HFI reached its highest peak with an incidence of almost 4 new cases. After this point, the hay fever slowly decreased with the temperature.

The second factor associating the HFI in a statistically significant manner was the wind speed (Figure 8). The wind speed had a bell-shaped association with HFI, where the curve direction changed at the middle. The HFI increased and reached its highest point at 3.2 new cases as the wind speed was in the range from 2.0 to 2.1 m/s. Beyond this point, the HFI decreased.

## 4 Discussion

A causal association between pollen and hay fever is well known. In general, pollen season is going to get longer and more intense with climate change (62–65), including alder, hazel, ash (66), birch, oak and pine (67), or herbaceous plants like grasses (68, 69) and ragweed (70). However, some other published literature showed no trends (71), or even decreasing trends (66, 72). The magnitude of flowering-intensity trends varies depending on the species, the geographical area, the flowering season, and climatic trends (73). In Germany, most hay fever cases are caused by eight different plant species. Hazel, alder, and birch from the *Betulaceae* (74, 75), ash (74, 76), grasses, rye, mugwort and ragweed (77). Air pollen concentrations of these plants are currently reported highest during Q2 and Q3.

Temperature is well known to affect pollen seasons (77–81), and in our study, this climate indicator was significantly associated with the HFI. Within the typical temperature range in Q1, the HFI rapidly increased and reached its first peak, which could be due to the effect of early bloomers' pollen, i.e. hazel and alder. This finding is important for the community in Freiburg for choosing the right therapeutic approach in patient treatment, since the symptoms for the common cold or flu can easily be mistaken with hay fever during this quarter. The second, more pronounced peak in HFI observed at ambient temperatures between 8°C and 13°C is likely attributable to increased exposure to mid-spring pollens, particularly from species such as ash and birch, as this temperature range coincides with their peak pollination period, typically occurring during the transition from the first to the second quarter of the year. The third peak could reflect the effect of the late flowers, i.e. grasses, rye, mugwort, and ragweed on the HFI. As we observed a significant increase in average temperature in Freiburg in Q3 and Q4 and its warmer-in-the-future tendency, the pollen season could be expected to start earlier in the future for trees that flower in late winter or early spring, such as hazel, alder, birch, and ash. For grasses, rye, mugwort, and ragweed, an extension of the vegetation period is to be expected in Freiburg, thus continuous pollen production later in the year may occur in the future. This assumption aligns with the results by Bergmann et al., that species advanced their pollen season more in early spring (e.g., hazel and alder by up to 2 days per year), than in mid spring (birch, ash) (82). A study from Poland demonstrated that grasses' pollen seasons lengthened by 2 to nearly 4 days between 1996 and 2011, which is related to warmer summer temperatures and later pollen season end dates (83). Zhang and Steiner predicted, that in the future warmer end-of-century temperatures will advance the onset of spring emissions by 10–40 days, and delay the onset of summer/fall emissions from weeds and grasses by 5–15 days, also extend the season length (84). Furthermore, Ziska et al. illustrated, that ragweed plants in America grew faster, flowered earlier, produced more pollen in cities than in rural sites, and persisted longer until the plants die in the first frost (85). The pollen season's prolongation could in turn lead to an increase in the pollen's total amount in the air, increasing the risk for hay fever sufferers.

Higher fall temperatures could lead to more unpredictable and extreme weather, e.g. storms, which could also have serious consequences for hay fever sufferers. Pollen grains can be carried along by thunderstorms on the ground, and the less resistant inner part of the pollen could be released into atmosphere and trigger additional allergic reactions (86).

Based on observed temperature trends over the past 15 years, it is plausible that the timing of new hay fever cases in Freiburg may shift from Q2 toward Q1 and Q4, potentially due to changes in pollen dynamics. Consequently, the hay fever season could extend into the winter months, increase in summer, and potentially become more evenly distributed throughout the year. However, this remains a hypothesis that requires further investigation. Our current data do not allow for definitive conclusions regarding future temporal shifts in HFI. Longitudinal monitoring of both climate indicators and aeroallergen concentrations, coupled with predictive modeling, will be essential to confirm these potential trends. Future studies incorporating fine-scale phenological data and patient-level exposure assessments could provide a clearer understanding of how climate change may alter the seasonal distribution of hay fever cases.

The second factor showing a significant relationship with the hay fever was the wind speed. A study by Damialis et al. indicated that pollen atmospheric movement is a very complex phenomenon influenced by numerous environmental parameters with wind playing a major role (87). The positive association in our study's result was indirectly in line with previous studies, indicating that higher wind speeds reduce pollen grain mass via dehydration, enhancing atmospheric dispersal (88). In addition, pollen that has already been settled can be pushed upwards in the atmosphere by the action of the wind (89). Interestingly, as the wind speed exceeded 2.0 m/s, the HFI started decreasing, indicating a negative association between the HFI and high wind speed in this study. This aligns with findings by Wang et al. (17). One possible explanation for such an event is provided by Niklas (90) and Oh (91) that the wind decreases the amount of local pollen by carrying local pollen to other places. No significant variation of the wind speed was observed over the last 15 years in Freiburg. Thus, the same association between the HFI and the wind speed is anticipated in the future.

Until now, very few studies have investigated the association between hay fever and precipitation, and only limited number supported the association (17). Whereas several published literatures suggested a relationship between precipitation and pollen in relation to the slowdown in photosynthesis (92–94), the wash-out effect (95), the thunderstorm's effects (96), the enhanced growth of trees/plants after getting nitrates in rainwater and the leaf wetting effect (93). Additionally, precipitation is also related to the increase of fungal spore (97). Nevertheless, these are just short-lived effects, which is difficult to capture by our quarterly data.

Our insignificant relations between the HFI and the concentrations of PM_10_, NO_2_, O_3_ in Freiburg align with several studies (27, 39, 98–100), but do not support some others. Regarding PM_10_, associations have been identified between PM_10_ and hay fever severity (35), hay fever prevalence (101). Specially, PM_10_ may act as carrier of adsorbed allergens and cause allergic reactions (102). Regarding NO_2_, a significant association with hay fever prevalence was found by Teng et al. (101). In animal tests, negative effects of NO_2_ on respiratory system and severity of allergic airway inflammation have been also demonstrated (37, 38). Additionally, effects of NO_2_ on various pollen allergens have been assumed (33, 34, 90, 93, 103, 104), which may then affect hay fever sufferers. Interestingly, only few studies indicated that ground-level O_3_ increases hay fever risk (23, 105–107). Furthermore, individual O_3_ molecules can cling to pollen surfaces and enter the airway (108). O_3_ is also an important stress factor for plants, which can damage plant cells, trigger them to discharge more pollen (109), and alter allergenicity of pollen in both negative and positive manners (110–113). However, all these results were derived for high concentrations of the environmental stressors, whereas the quarterly concentrations of PM_10_, NO_2_ and O_3_ in Freiburg across the study years exhibited comparably low levels. This could account for the insignificant findings reported in our study.

Several limitations of our study have to be reported: the hay fever data was sampled quarterly, which may not capture short-term fluctuations in hay fever that occur due to changes in environmental factors. When data are aggregated over a long period, important information about short-term changes get lost. Another shortcoming of our dataset is that hay fever data have been reported as incidences, which only reflect the number of new cases developed within the investigated time, but does not provide information about the severity or duration of symptoms experienced by new diagnosed and already affected patients. Moreover, a longer time series of data may lead to better statistical results in what regards the accuracy and precision of the effects measured. The coronavirus pandemic in 2020 to 2022 had given rise to social distancing and self-isolation. This might have led to a decline in the number of doctor visits and fewer diagnosed cases in consequence. It may also have resulted in fewer opportunities for children and adolescents to be infected with respiratory and other communicable diseases. This, in turn, may also have affected the numbers of HFI during this time period. The German Statutory Health Insurance is a group of public health insurance, which covers most of the working classes. Data from privately insured people, who potentially have higher living standards, were not covered. This group also needs to be investigated in the future. The stationary data may not fully capture the variations of the climate indicators due to personal microclimate and mobility, which is difficult to measure so far. Future studies should aim at more individualized information regarding the microclimate and mobility. Finally, the absence of pollen concentration data is a significant limitation in our study, as pollen is typically the primary trigger for hay fever, with climate factors as proxies. Future research investigating associations with hay fever should also include the pollen's contribution in the analysis.

## 5 Conclusion

Our findings could not support the associations between the HFI and the precipitation as well as the concentrations of PM_10_, NO_2_, and O_3_ in Freiburg. However, the results for the temperature and the wind speed in relation to the HFI were highlighted. Here, the HFI was highest when the temperature was between 4–6°C and 10–17°C, and the wind speed was between 2.0 and 2.1 m/s. Thus, awareness of local environmental conditions may assist healthcare providers in recognizing patterns of allergen exposure that coincide with peaks in hay fever symptoms. While direct monitoring of meteorological data is not expected in routine clinical practice, integrating local environmental health advisories and pollen forecasts into clinical awareness could improve diagnostic accuracy. Such an approach may facilitate earlier initiation of targeted therapies for allergic rhinitis, potentially improving patient outcomes.

## Data Availability

The data analyzed in this study is subject to the following licenses/restrictions: the dataset is available on request from the corresponding author. Requests to access these datasets should be directed to evelyn.lamy@med.uni-augsburg.de.
