# Supplementary material for: Association between climate indicators and hay fever incidence in children and adolescents in Freiburg, Germany
Source: Front Public Health. 2025 Jun 4;13:1587767. doi: 10.3389/fpubh.2025.1587767 (PMC12174111; doi:10.3389/fpubh.2025.1587767)
Supplement: Supplementary file 1 [file Table_1.docx]

**S2: Demographic Information of the population at risk of hay fever**

| **Year** | **Age Groups (children and adolescents)** | | | | | **Sex (%)** | |
| --- | --- | --- | --- | --- | --- | --- | --- |
|  | **0-2** | **3-6** | **7-10** | **11-13** | **14-17** | **Male** | **Female** |
| **2013** | 5268 | 5376 | 4767 | 3218 | 4485 | 50.818 | 49.182 |
| **2014** | 5466 | 5439 | 4785 | 3148 | 4558 | 50.859 | 49.141 |
| **2015** | 5540 | 5546 | 4852 | 3317 | 4405 | 50.752 | 49.248 |
| **2016** | 5725 | 5657 | 4816 | 3353 | 4334 | 50.517 | 49.483 |
| **2017** | 6029 | 5708 | 4907 | 3317 | 4319 | 50.395 | 49.605 |
| **2018** | 6127 | 5885 | 5171 | 3445 | 4376 | 50.468 | 49.532 |
| **2019** | 6177 | 6102 | 5345 | 3508 | 4568 | 50.576 | 49.424 |
| **2020** | 6251 | 6508 | 5550 | 3570 | 4731 | 50.684 | 49.316 |
| **2021** | 6215 | 6622 | 5526 | 3824 | 4862 | 50.715 | 49.285 |
